# Tracing Visual Expertise in ECG Interpretation: An Eye‐Tracking Pilot Study

**DOI:** 10.1111/anec.70082

**Published:** 2025-04-18

**Authors:** Alessandro Bortolotti, Fabrizio Ricci, Carmelita Cieri, Federica Cocco, Chiara Martini, Marcello Panunzi, Davide Rossi, Anna Sorella, Silvio Saraullo, Davide Scordo, Giulia Renda, Sabina Gallina, Riccardo Palumbo

**Affiliations:** ^1^ Department of Neuroscience, Imaging and Clinical Sciences G. D'annunzio University of Chieti‐Pescara Chieti Italy; ^2^ University Cardiology Division, Heart Department Policlinico SS. Annunziata Chieti Italy; ^3^ Department of Clinical Sciences Lund University Malmö Sweden; ^4^ Institute for Advanced Biomedical Technologies, G. D'annunzio University of Chieti‐Pescara Chieti Italy

**Keywords:** acute coronary syndrome, electrocardiogram interpretation, emergency departmentcardiology education, eye‐tracking, visual expertise

## Abstract

**Background:**

Visual expertise is pivotal for accurate ECG interpretation. We aimed to identify and measure expertise‐based differences in visual search patterns, cognitive load, and diagnostic accuracy during ECG analysis using eye‐tracking technology.

**Methods:**

First‐ to third‐year residents and board‐certified expert cardiologists interpreted ECGs of patients with suspected acute coronary syndrome, while eye‐tracking glasses recorded fixation count, duration, and pupil dilation. Diagnostic accuracy and cognitive load via NASA Task Load Index were analyzed. Heatmaps illustrated relationships between cognitive load, perceived workload, and self‐assessed performance across experience levels and ECG task complexities.

**Results:**

Expert readers interpreted ECGs significantly faster than residents (107.6 ± 32.8 vs. 205.31 ± 57.43 s; *p* < 0.001) and demonstrated higher diagnostic accuracy across all levels of task difficulty (*p* < 0.001). Eye‐tracking analysis revealed that experts exhibited fewer fixations (67.7 ± 25.7 vs. 143.7 ± 29.9; *p* < 0.001) and longer fixation durations (3.9 ± 0.7 vs. 3.2 ± 1 s; *p* = 0.032) than residents. Experts also showed lower pupil dilation changes (4.8% ± 2% vs. 10.5% ± 4.2%; *p* = 0.015). Increased task difficulty was associated with greater pupil dilation, particularly among novices (mean pupil dilation for difficult tasks 13.4% ± 4.1% vs. 7.3% ± 2.3% for easy tasks; *p* = 0.008), indicating higher cognitive demand. Experts maintained superior self‐assessed performance (8 ± 0 vs. 7 ± 1.2; *p* = 0.009) and reported lower perceived negative workload (4.5 ± 1.45 vs. 6 ± 0.55; *p* = 0.041).

**Conclusions:**

In this pilot study, expert readers achieved faster and more accurate diagnoses, exhibiting more efficient visual search patterns and lower cognitive load. Pending external validation, our findings suggest that ECG training programs should focus on developing targeted visual techniques, cognitive efficiency, and adaptive coping strategies to enhance accurate interpretation.

## Graphical abstract

The study examines how clinical experience affects the interpretation of ECG using eye‐tracking metrics to analyze visual search patterns and cognitive load. The results show that experts interpret ECGs more quickly and accurately than residents, thanks to more efficient visual strategies and lower cognitive load. These findings suggest that experience improves diagnostic efficiency and cognitive resource management, with significant implications for medical training.

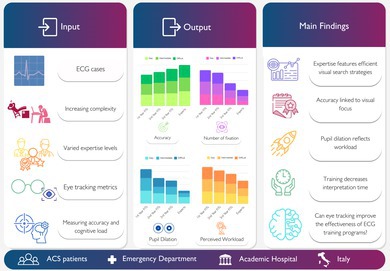

## 1 Introduction

The interpretation of electrocardiograms (ECGs) is a cornerstone of cardiovascular medicine, providing crucial diagnostic insights that guide the management of a wide range of cardiac conditions (Fanaroff et al. 2018; Salerno et al. 2003; Schläpfer and Wellens 2017). Within the high‐pressure environment of the emergency department (ED), rapid and accurate ECG interpretation is especially vital for the timely identification and treatment of acute coronary syndromes (ACS) (Benjamin et al. 2019; Wathen et al. 2019). ACS, which includes conditions such as ST‐elevation myocardial infarction (STEMI), non‐ST elevation myocardial infarction (NSTEMI), and unstable angina, remains a leading cause of morbidity and mortality globally (Novotny et al. 2020; Thygesen et al. 2018; Zègre‐Hemsey et al. 2012).

Despite the fundamental role of ECG interpretation in modern cardiology, achieving proficiency in this skill is challenging. The complexity of interpreting ECGs lies in the need to rapidly synthesize visual information into a coherent clinical picture while managing the cognitive demands of time‐sensitive decision‐making (Gegenfurtner et al. 2011; Sibbald et al. 2013; Waechter et al. 2019). For trainees, from medical students to junior cardiology residents, this often results in a steep learning curve, where inexperience can lead to missed diagnoses or delayed interventions (Bond et al. 2014; Breen et al. 2014; Wood et al. 2013).

Visual expertise in ECG interpretation is more than a matter of accumulating knowledge; it involves the development of refined cognitive strategies that allow clinicians to focus on diagnostically relevant elements of the ECG while minimizing distractions (Ericsson 2004; Rosen et al. 2018; Wathen et al. 2019). Expert interpreters, through years of clinical practice, appear to develop an intuitive ability to recognize critical patterns, a skill that allows them to quickly identify abnormalities even in the presence of complex or atypical presentations (Qiao et al. 2018; Wood et al. 2016).

Previous studies have suggested that this proficiency is supported by specific visual search behaviors, where experts demonstrate a more focused and efficient scanning of ECG waveforms compared to novices (Coderre et al. 2010; Kok et al. 2016). However, the cognitive underpinnings of this process, including how experts manage cognitive load during interpretation, remain poorly characterized (Hart and Staveland 1988; Wood et al. 2013).

## 2 Methods

### 2.1 Study Design and Setting

This retrospective, observational investigation was conducted in a leading academic hospital setting, leveraging a carefully curated set of nine ECGs obtained from patients presenting with acute chest pain and suspected acute coronary syndrome (ACS). These cases were selected to reflect a broad spectrum of clinical scenarios, including classic presentations of ST‐elevation myocardial infarction (STEMI), nuanced cases of non‐ST elevation myocardial infarction (NSTEMI), and other noncoronary cardiac and noncardiac etiologies of chest pain (Salerno et al. 2003; Fanaroff et al. 2018).

### 2.2 Participants

A diverse cohort of eight participants was recruited, reflecting a continuum of clinical expertise in cardiology. These individuals were stratified into four groups of two: first‐year cardiology residents, second‐year cardiology residents, third‐year cardiology residents, and board‐certified cardiologists with extensive experience. This stratification allowed for a granular analysis of how interpretative skills evolve with training, from the early stages of residency to the level of board‐certified expert cardiologists (Benjamin et al. 2019; Wathen et al. 2019; Novotny et al. 2020).

### 2.3 Procedure

Interpretations were performed using the Tobii Pro Glasses 2, a state‐of‐the‐art wearable eye‐tracking device capable of capturing real‐time visual search patterns with precision. Participants were tasked with interpreting each of the nine ECGs within a strict five‐minute timeframe, mirroring the time‐pressured decision‐making environment of the ED. This time constraint was designed to emulate the urgency with which clinicians must often operate when triaging patients with potentially life‐threatening conditions (Thygesen et al. 2018; Zègre‐Hemsey et al. 2012).

Participants approached each ECG as they would in clinical practice, prioritizing the identification of key features such as ST‐segment deviations, T‐wave inversions, arrhythmic patterns, and other abnormalities indicative of ischemic or nonischemic etiologies. Following each interpretation session, participants completed the NASA Task Load Index (NASA TLX) questionnaire, a validated tool that provided a multidimensional assessment of perceived cognitive workload (Gegenfurtner et al. 2011; Sibbald et al. 2013; Waechter et al. 2019). This index included critical aspects such as mental demand, time pressure, and frustration—elements that directly impact diagnostic accuracy and decision‐making under stress (Bond et al. 2014; Breen et al. 2014; Wood et al. 2013).

### 2.4 Data Collection and Eye‐Tracking Analysis

The primary data comprised eye‐tracking metrics—fixation count, fixation duration, and pupil dilation—analyzed using the advanced Tobii Pro Lab software. Fixation count provided a quantitative measure of the number of visual engagements with distinct regions of the ECG, offering insight into the breadth of the search strategy (Ericsson 2004; Rosen et al. 2018; Wathen et al. 2019). Fixation duration captured the depth of focus, revealing the time spent analyzing critical segments of the ECG waveform (Qiao et al. 2018; Wood et al. 2016). Pupil dilation, a well‐established proxy for cognitive load, allowed us to infer the mental effort exerted during each interpretative task, with larger dilations correlating with higher cognitive demands (Coderre et al. 2010; Kok et al. 2016).

To complement these objective measures, interpretation accuracy was rigorously evaluated. Two senior cardiologists, blinded to participant identity, independently scored each ECG interpretation on a standardized scale from 1 (least accurate) to 5 (most accurate), ensuring a robust assessment of diagnostic performance. Any discrepancies between reviewers were resolved through consensus discussion, thus upholding the integrity of the scoring process (Hart and Staveland 1988; Wood et al. 2013).

### 2.5 Data Analysis

Statistical analysis was conducted to discern differences in interpretation accuracy, eye‐tracking metrics, and cognitive load across varying levels of experience. Descriptive statistics, including means and standard deviations, provided a foundational understanding of performance metrics within each group. ANOVA was employed to evaluate both intergroup differences and the impact of ECG difficulty on interpretation behaviors. This method allowed for a nuanced understanding of how both experience and task complexity shape visual search strategies (Gegenfurtner et al. 2011; Sibbald et al. 2013; Waechter et al. 2019).

In addition, Pearson correlation analyses were performed to examine the relationship between pupil dilation and interpretation accuracy, shedding light on the intricate interplay between cognitive load and diagnostic precision. All statistical analyses were performed using the R software (version 4.1.2). A significance threshold of *p* < 0.05 was applied (Bond et al. 2014; Breen et al. 2014; Wood et al. 2013).

## 3 Results

### 3.1 Participant Demographics and Task Characteristics

Eight cardiology practitioners, ranging from first‐year residents to board‐certified experts, participated in the study. Participants interpreted ECGs from ED patients with acute chest pain with ECGs categorized as easy, intermediate, or difficult based on clinical complexity (Tables 1 and 2, Supporting Information).

**TABLE 1:** Profile of ECG readers.

Gender	Age	Experience
Male	28	First year FIT
Female	25	First year FIT
Male	29	Second year FIT
Female	27	Second year FIT
Male	32	Third year FIT
Female	28	Third year FIT
Male	39	Expert
Female	52	Expert

**TABLE 2:** ECG case details.

Task	Difficulty	ECG report	Final diagnosis
ECG1	Easy	Sinus rhythm with a heart rate of 79 bpm, ST‐segment elevation in leads II, III, aVF, and V1‐V	STEMI
ECG2	Intermediate	Atrial fibrillation, heart rate 111 bpm, left anterior fascicular block + right bundle branch block, subtle ST‐segment changes	NSTEMI‐OMI
ECG3	Easy	Sinus rhythm with heart rate of 74 bpm, no repolarisation abnormalities	Noncardiac chest pain
ECG4	Difficult	Sinus rhythm with heart rate of 83 bpm, negative T waves in V2‐V4	NSTEMI‐OMI
ECG5	Easy	Sinus rhythm with heart rate of 67 bpm, hyperacute T waves in anterior leads	STEMI
ECG6	Difficult	Sinus rhythm with heart rate of 90 bpm, ST‐segment depression in leads V4‐V6.	NSTEMI‐OMI
ECG7	Intermediate	Sinus rhythm with heart rate of 81 bpm, left bundle branch block	Heart failure exacerbation
ECG8	Intermediate	Sinus rhythm with heart rate of 77 bpm, minimal ST‐segment changes in lateral leads	NSTEMI‐OMI
ECG9	Difficult	Sinus rhythm with heart rate of 72 bpm, deep ST‐segment depressions in precordial leads	NSTEMI‐OMI

### 3.2 Interpretation Time and Diagnostic Accuracy

**FIGURE 1:**
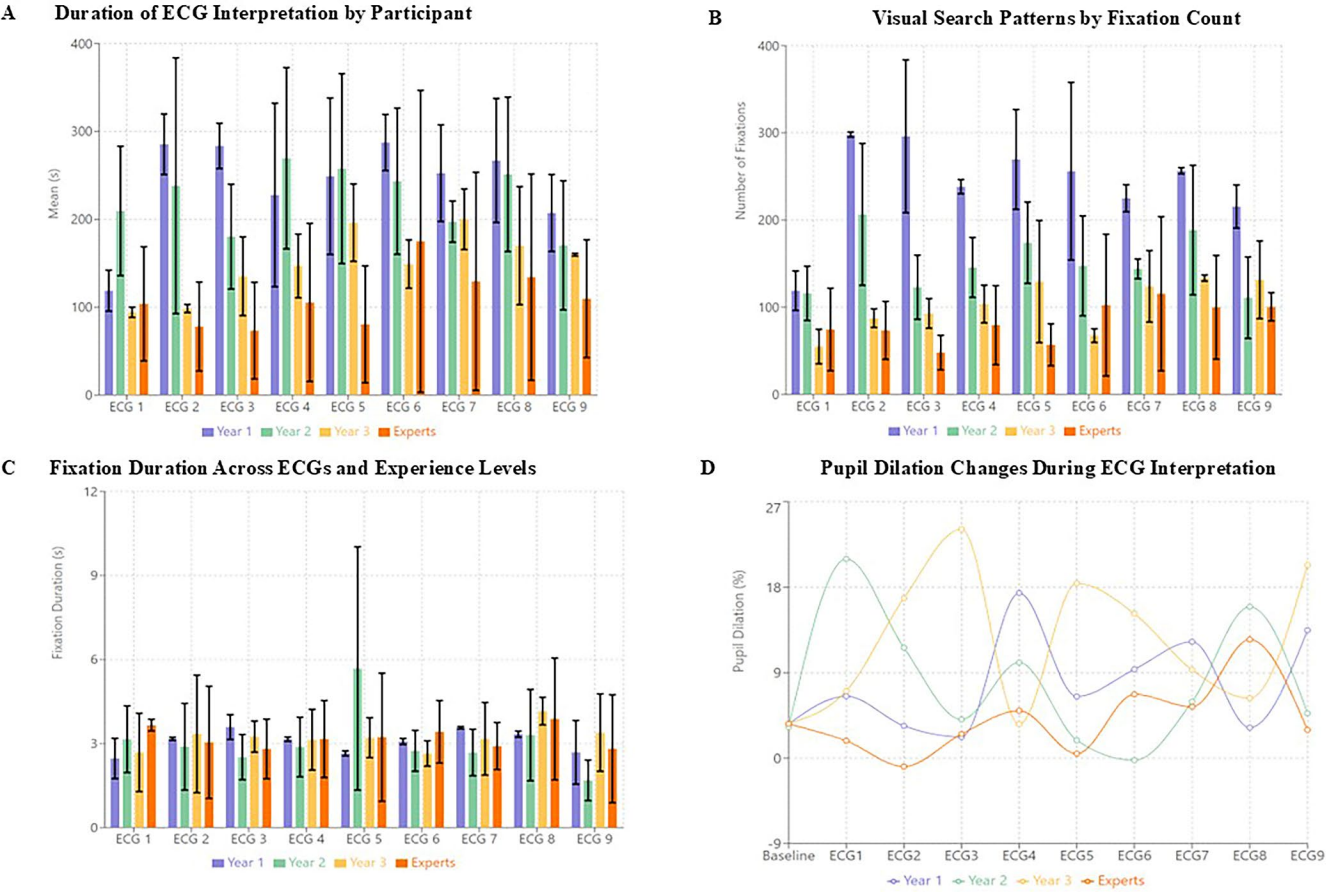
ECG Interpretation and Eye‐Tracking Metrics Across Experience Levels. (A) illustrates the time taken by participants from different experience levels to interpret each of the nine ECGs. The bars represent the mean interpretation time, with error bars indicating the variability (standard deviation). Experts consistently demonstrate shorter interpretation times compared to less experienced participants, highlighting the role of expertise in rapid decision‐making. (B) shows the number of fixations made by participants across different ECGs, segmented by experience level. Higher fixation counts among less experienced participants indicate a more scattered and exhaustive visual search strategy, while experts show fewer fixations, suggesting a more targeted and efficient approach to identifying key ECG features. (C) displays the average duration of fixations across different ECGs for each experience group. Longer fixation durations among experts suggest a deliberate focus on critical regions, whereas shorter and more variable fixations among novices reflect uncertainty and a need for reexamination. Error bars represent the variability (standard deviation) in fixation duration. (D) shows the changes in pupil dilation as a marker of cognitive load across different ECGs and experience levels. Greater pupil dilation in younger participants suggests higher cognitive effort, particularly during complex ECG interpretation. Experts show smaller fluctuations in pupil dilation, indicative of reduced cognitive strain and familiarity with the task. The baseline represents the resting pupil size before interpretation.

Experts interpreted ECGs significantly faster than residents (mean time, 107.61 ± 32.78 s vs. 205.31 ± 57.43 s; *p* < 0.001). Interpretation times decreased with increasing levels of training, with first‐year residents exhibiting the longest times and experts showing the shortest, consistent across all ECG difficulty levels (Figure 1). Diagnostic accuracy was also higher among experts compared to residents (*p* < 0.001), with experts maintaining a consistently high level of accuracy across varying task complexities. Differences in accuracy were less pronounced in simpler ECGs but increased markedly with task difficulty (Table 3).

**TABLE 3:** Comparative summary of key metrics of ECG interpretation proficiency, eye‐tracking metrics, and perceived workload.

ECG analysis	1st year FITs (Mean ± SD)	2nd year FITs (Mean ± SD)	3rd year FITs (Mean ± SD)	Experts (Mean ± SD)	*p*	Interpretation
ECG report grading	3.67 ± 0.90	3.72 ± 0.85	4.22 ± 0.75	4.67 ± 0.43	< 0.001	Expertise tracks greater proficiency and consistency in ECG interpretation
Diagnostic accuracy, %	73.33 ± 4.34	77.77 ± 3.85	84.44 ± 6.16	93.33 ± 9.42	< 0.001	Significant increase in accuracy with experience
Interpretation time, s	241.96 ± 59.51	224.08 ± 34.45	149.90 ± 37.01	107.61 ± 32.78	< 0.001	Experts interpret ECGs significantly faster
Eye tracking						
Number of Fixations	179.41 ± 28.67	157.32 ± 36.91	94.25 ± 22.10	67.68 ± 25.74	< 0.001	Decrease in fixation number as expertise increases
Fixation uration, s	2.95 ± 0.58	3.13 ± 1.02	3.56 ± 1.12	3.87 ± 0.71	0.032	Longer fixation durations observed in experts, indicating focused attention
Pupil dilation, %	10.33 ± 3.56	8.45 ± 4.02	12.79 ± 5.14	4.83 ± 2.01	0.015	Experts exhibit smaller changes, indicating lower cognitive load
NASA‐TLX Load Index						
Performance	9.00 ± 0.00	5.50 ± 0.71	7.50 ± 0.71	8.00 ± 0.00	0.009	Performance improves with increased experience, reflecting better task proficiency in experts
Mental demand	6.50 ± 0.71	7.00 ± 0.00	6.50 ± 2.12	5.50 ± 0.71	0.661	No significant difference across groups, suggesting similar mental processing effort regardless of expertise level
Physical demand	5.00 ± 0.00	4.00 ± 4.24	5.00 ± 0.00	3.00 ± 1.41	0.615	Physical demand does not vary significantly, implying it is not a major differentiator between experience levels
Temporal demand	5.00 ± 0.00	8.50 ± 0.71	6.00 ± 1.41	6.50 ± 0.71	0.062	Temporal demand peaks in 2nd year FITs, potentially due to a transitional phase of learning; experts manage time more efficiently
Effort	4.50 ± 4.95	8.00 ± 0.00	6.50 ± 0.71	5.50 ± 2.12	0.383	Effort levels are consistent across groups, indicating a steady exertion level regardless of expertise
Frustration	2.50 ± 0.71	8.50 ± 0.71	4.00 ± 1.41	4.50 ± 0.71	0.012	Frustration is significantly higher among 2nd year FITs, possibly due to increased task complexity during intermediate training stages
Negative domains	6.40 ± 1.95	6.00 ± 0.95	5.60 ± 1.36	4.50 ± 1.45	0.361	No significant trend observed; however, a slight reduction in negative experiences is seen with growing expertise

*Note:* The table illustrates the progression of skills from 1st year Fellows‐in‐Training (FITs) to experts, highlighting aspects of ECG interpretation proficiency eye‐tracking metrics, and perceived workload by NASA Task Load Index scores. Significant improvements are noted in accuracy and speed among experts, accompanied by reduced cognitive load, as indicated by fewer fixations and smaller pupil dilation. Performance trends also suggest increased proficiency with experience, while intermediate levels show heightened temporal demand and frustration.

### 3.3 Visual Search Patterns

Eye‐tracking metrics highlighted distinct differences in visual search behavior. Experts exhibited fewer fixations per ECG compared to residents (mean 67.68 ± 25.74 vs. 143.66 ± 29.89; *p* < 0.001), indicating a more streamlined visual approach. Fixation duration was also longer in experts (mean 3.87 ± 0.71 s vs. 3.21 ± 0.96 s for residents; *p* = 0.032). These trends were consistent across all levels of ECG complexity (Figure 1). Notably, residents showed greater variability in fixation patterns, with increased fixations observed during more challenging cases.

### 3.4 Pupil Dilation and Cognitive Load

**FIGURE 2:**
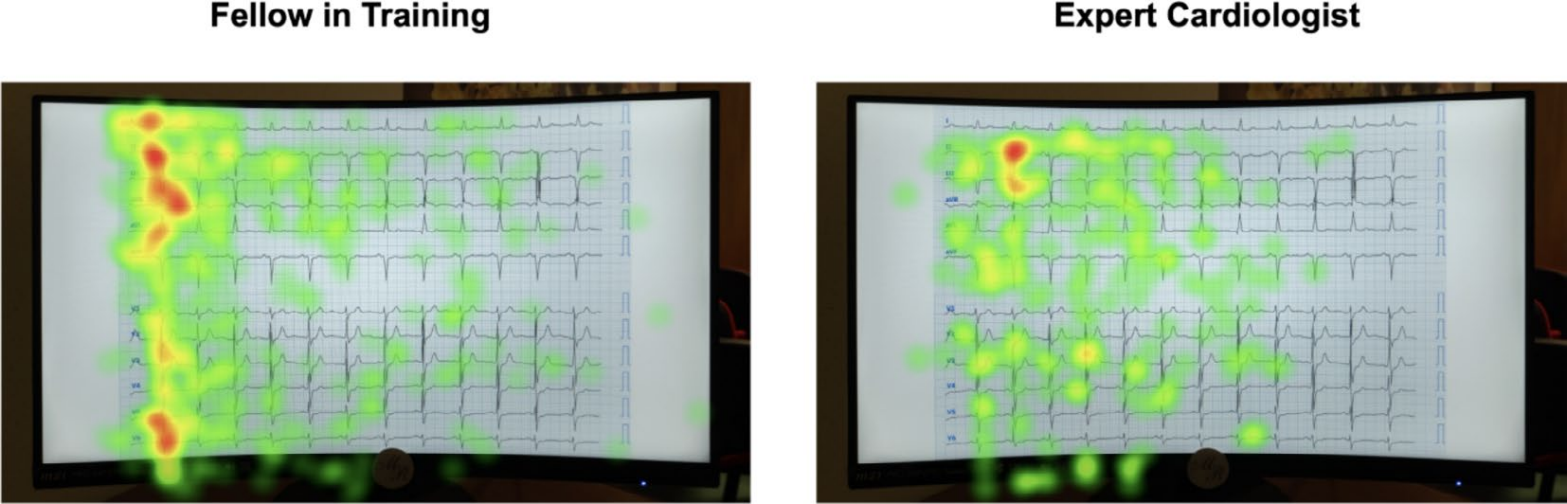
Visual search patterns in novice versus expert ECG readers. This figure illustrates the differences in visual search strategies between a fellow in training (left) and an expert (right) during the interpretation of ECG 6. The heat maps represent areas of concentrated visual attention, with warmer colors (red and yellow) indicating regions where participants focused most intensely. The novice's heat map shows a dispersed pattern of attention, with a significant focus on nondiagnostic regions, suggesting an exhaustive search strategy. In contrast, the expert's heat map reveals a more targeted approach, with concentrated fixations on diagnostically relevant areas, such as the ST‐segment and T‐wave regions. This difference in visual focus underscores the efficiency of expert interpreters in identifying critical ECG features with reduced cognitive effort.

Pupil dilation data provided insights into cognitive load during ECG interpretation. Experts displayed lower changes in pupil dilation (mean 4.83% ± 2.01% vs. 10.52% ± 4.18% for residents; *p* = 0.015), indicating reduced cognitive strain. Across all participants, increased task difficulty was associated with greater pupil dilation, particularly among less experienced readers (mean dilation for difficult tasks: 13.45% vs. 7.32% for easy tasks; *p* = 0.008) (Figures 1 and 2). These findings were consistent across different groups, with experts showing minimal fluctuation in pupil dilation even during the most complex cases, whereas novices exhibited marked increases in dilation.

### 3.5 Self‐Assessed Performance and Perceived Workload

**FIGURE 3:**
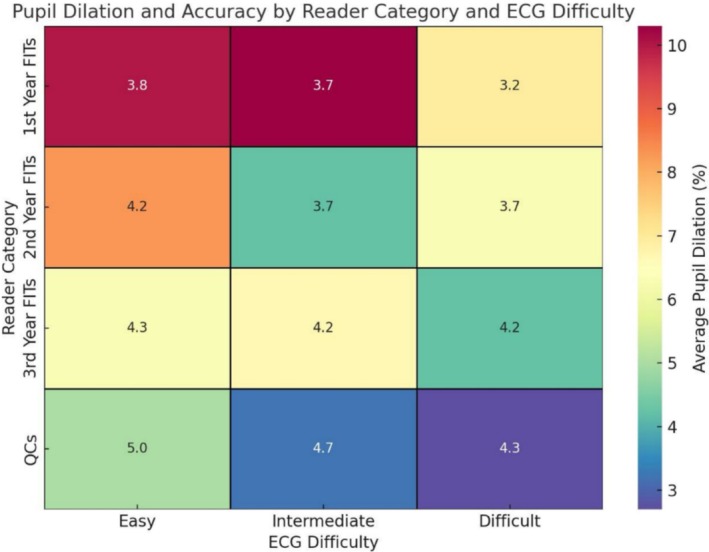
Heatmap analysis of pupil dilation and ECG interpretation accuracy. The heatmap provides a visual analysis of the relationship between average pupil dilation and interpretation accuracy across different reader categories and ECG task difficulties. The color intensity represents the average pupil dilation, with cooler tones (blue/green) indicating lower dilation and warmer tones (yellow/red) signifying higher dilation. This allows for an intuitive understanding of how cognitive load, as measured by pupil dilation, changes depending on the difficulty of the ECG interpretation tasks. The *X*‐axis categorizes the ECG tasks into three difficulty levels: Easy, Intermediate, and Difficult. Each column represents the average pupil dilation for ECGs within that difficulty group. The *Y*‐axis distinguishes between reader categories, from 1st Year FITs (fellows in training) to QCs (qualified cardiologists), indicating different levels of experience and expertise. Inside each cell, the numerical annotation denotes the average score of report quality for the reader group corresponding to the specific ECG difficulty. Accuracy scores range from 0 to 5, with 5 being the highest level of accuracy. This dual representation enables a simultaneous assessment of how both cognitive load and interpretation accuracy vary across different levels of expertise and task challenges. By examining the patterns, one can discern whether higher task difficulty correlates with increased pupil dilation, potentially indicating greater cognitive effort. Similarly, the accuracy scores offer insights into how well different reader groups perform under varying levels of task difficulty, which may inform training and assessment practices.

**FIGURE 4:**
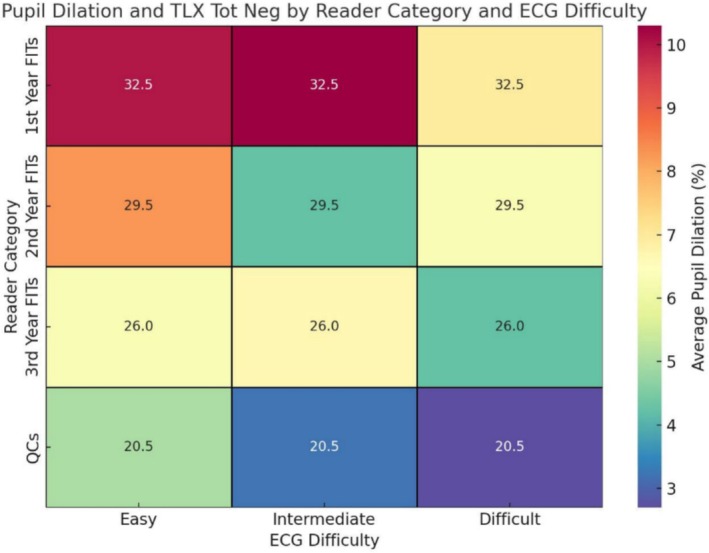
Heatmap analysis of pupil dilation and perceived workload. The heatmap visualizes the relationship between cognitive load, measured by average pupil dilation (%), and perceived task difficulty, indicated by “TLX_Tot_Neg” scores from the NASA Task Load Index (TLX). Color intensity reflects pupil dilation, with warmer tones (yellow/red) indicating higher cognitive load (greater dilation) and cooler tones (blue/green) representing lower load (less dilation). Numerical values within each cell represent average TLX scores, where higher scores suggest a greater perceived negative workload. The *X*‐axis categorizes ECG tasks by difficulty level (Easy, Intermediate, Difficult), facilitating a clear comparison across different task complexities. The *Y*‐axis organizes participants based on their training and expertise: 1st Year FITs, 2nd Year FITs, 3rd Year FITs, and experienced QCs. This comprehensive view illustrates how different reader groups respond to varying task complexities, both physiologically (through dilation) and subjectively (via TLX scores), providing insights into how experience influences cognitive and perceived workload.

**FIGURE 5:**
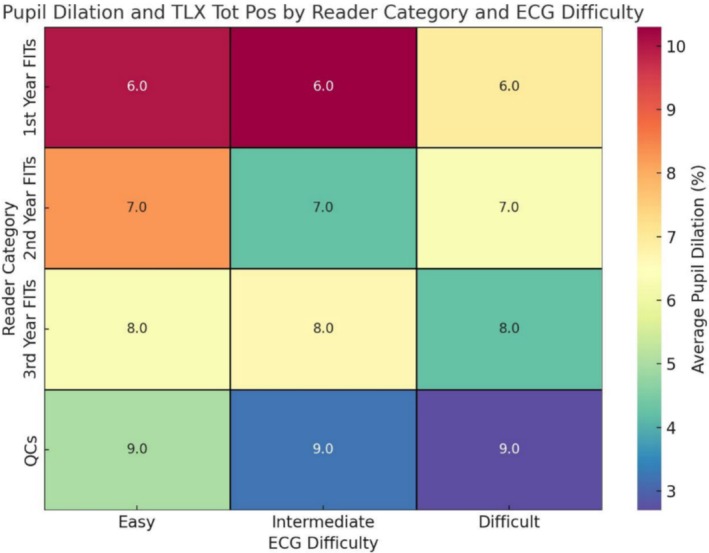
Heatmap analysis of pupil dilation and self‐assessed performance. The heatmap illustrates the relationship between cognitive load, as measured by average pupil dilation (%), and self‐assessed performance, captured through “TLX_Tot_Pos” scores from the NASA Task Load Index (TLX). Color intensity reflects pupil dilation, with warmer tones (yellow/red) indicating higher cognitive load (greater dilation) and cooler tones (blue/green) representing lower load (less dilation). Numerical values within each cell correspond to the average “TLX_Tot_Pos” scores, with higher scores indicating better perceived performance. The *X*‐axis organizes ECG tasks by difficulty (Easy, Intermediate, Difficult), enabling straightforward comparisons across varying complexities. The *Y*‐axis categorizes participants into reader groups: 1st Year FITs, 2nd Year FITs, 3rd Year FITs, and QCs, representing increasing levels of experience and expertise. This visualization highlights the interplay between cognitive load and self‐assessed performance across reader categories and task difficulties, offering insights into how experience and task complexity affect both physiological and subjective measures of performance. Resource management, with significant implications for medical training.

Self‐assessment scores reflected participants' perceptions of their diagnostic performance and perceived workload. Experts rated their performance higher than residents across all ECG complexities (mean score 8.00 ± 0.0 vs. 7.00 ± 1.21; *p* = 0.009). Additionally, experts reported lower perceived negative workload (mean score 4.5 ± 1.45 vs. 6 ± 0.55 for residents; *p* = 0.041). Heatmap analyses further illustrated that while perceived workload increased with task difficulty for all groups, experts reported a more stable and lower overall workload compared to novices (Figures 3, 4, 5).

## 4 Discussion

This study offers critical insights into the intricate relationship between visual expertise and ECG interpretation within the emergency department setting, emphasizing the role of expertise in enhancing diagnostic accuracy, efficiency, and cognitive resource management. The integration of visual search and cognitive load metrics showed clear distinctions in expertise across all levels of ECG difficulty. Experts exhibited fewer fixations, longer fixation durations, and reduced pupil dilation, suggesting a more efficient and focused approach to ECG interpretation. In contrast, residents displayed greater pupil dilation, increased fixation counts, and shorter, scattered fixations, reflecting higher cognitive demands and less efficient diagnostic strategies. These patterns were particularly evident in more challenging ECGs, where the gap in performance and cognitive load between experts and residents widened significantly. Our data underscores a progressive improvement in ECG interpretation with increasing clinical experience, where experts not only deliver faster and more accurate interpretations but also exhibit a refined capacity to manage cognitive load, as indicated by eye‐tracking metrics (Salerno et al. 2003; Fanaroff et al. 2018; Benjamin et al. 2019; Schläpfer and Wellens 2017).

### 4.1 Efficiency and Diagnostic Accuracy

The significant differences in interpretation times and visual search patterns between novices and experts reflect the cognitive transition that accompanies skill acquisition. Novices rely heavily on a broad, exhaustive search strategy, which, while thorough, is time‐consuming and cognitively taxing. These patterns are clearly illustrated in the heat map analyses, where novices display a dispersed pattern of fixations with intense focus on nondiagnostic regions. Our findings demonstrate that expertise is closely associated with greater proficiency and consistency in ECG interpretation, as evidenced by higher ECG report grading scores and diagnostic accuracy. Across all levels of task difficulty, experts consistently achieved more accurate interpretations, which correlated with their ability to focus more effectively on relevant ECG features, such as specific leads or waveform segments, minimizing time spent on nonessential elements. This efficiency is likely a product of the extensive experience and pattern recognition skills that experts accumulate over time (Wathen et al. 2019; Novotny et al. 2020; Thygesen et al. 2018). Furthermore, experts interpreted ECGs significantly faster than residents, indicating that the speed of interpretation improves alongside accuracy, without compromising diagnostic quality. These trends suggest that experience drives both diagnostic efficiency and precision, which are critical for time‐sensitive decision‐making in the ED. This targeted attention is crucial for rapid decision‐making in time‐sensitive environments like the ED (Zègre‐Hemsey et al. 2012; Ericsson 2004; Rosen et al. 2018).

### 4.2 Visual Focus and Diagnostic Efficiency

The contrast between novice and expert heat maps highlights the underlying cognitive differences. Novices tend to explore a wider area, focusing heavily on sections like lead labels without a structured approach. This reflects their higher cognitive load and unfamiliarity with prioritizing key ECG features. Conversely, the expert's heat map shows a streamlined visual approach, with fixations concentrated over diagnostically important areas such as the ST‐segment and T‐wave regions. This efficiency likely results from extensive experience and the ability to recognize critical patterns with minimal effort. These findings suggest that the observed differences in fixation number and duration are not merely a matter of speed but represent a deeper shift in cognitive processing that develops with expertise (Qiao et al. 2018; Wood et al. 2016; Coderre et al. 2010).

### 4.3 Cognitive Load and Pupil Dilation

Pupil dilation data further reveals the cognitive ease with which experts navigate complex ECG interpretations. Reduced changes in pupil size among experts suggest a lower cognitive burden during interpretation, even in challenging cases. This aligns with theories of cognitive automation, where repetitive exposure to similar clinical scenarios allows experts to process complex information with minimal mental effort. In contrast, the heightened pupil dilation seen in less experienced interpreters indicates that they are working at or near their cognitive limits, reflecting a more deliberate and effortful process of information gathering and interpretation. These differences in cognitive load, as highlighted by pupil dilation metrics, complement the heat map findings by providing a quantitative measure of the mental effort exerted during ECG interpretation (Kok et al. 2016; Hart and Staveland 1988; Wood et al. 2013).

When comparing these findings to those of a similar study involving eye‐tracking during ECG interpretation, several consistent patterns emerge. That study also identified that more experienced interpreters tend to focus more directly on specific leads and abnormalities, relying on a targeted and efficient approach rather than the comprehensive scanning seen in less experienced participants. However, while both studies agree on the overall trend of decreasing fixation counts with increasing expertise, the present study goes further by emphasizing the role of cognitive load through pupil dilation metrics, providing a more detailed understanding of the mental effort required during ECG interpretation (Bond et al. 2014; Breen et al. 2014; Wood et al. 2013).

Moreover, while the other study highlighted the variability in fixations among different levels of practitioners, this study's inclusion of pupil dilation offers an additional layer of understanding, suggesting that experts' lower fixation counts are not simply a matter of speed but reflect a deeper cognitive economy. By quantifying how much cognitive effort each participant exerted, this study sheds light on the cognitive strategies that underpin expert performance, offering a more holistic perspective on the evolution of visual expertise (Ericsson 2004; Rosen et al. 2018; Wathen et al. 2019).

### 4.4 Implications for Training and Education

The heat map analysis vividly illustrates the difference between novice and expert approaches to ECG interpretation, reinforcing the value of experience in achieving a more efficient and effective diagnostic process. These findings underscore the importance of training programs that focus on helping novices develop more targeted visual strategies. By learning to focus their attention on diagnostically critical areas, novices can reduce their cognitive load and improve their interpretation speed and accuracy. Eye‐tracking feedback could be a valuable tool in this process, helping trainees visualize their own gaze patterns in comparison to those of experts and adjust their approach accordingly (Qiao et al. 2018; Wood et al. 2016; Coderre et al. 2010).

In particular, understanding the strategies that allow experts to balance speed with accuracy could inform the development of more effective educational interventions, tailored to accelerate the acquisition of these skills among trainees. This is especially crucial in the ED, where time pressures and the high stakes of cardiac care demand not only accuracy but also the ability to act decisively. By offering a detailed analysis of how expertise modifies the approach to ECG interpretation, this research seeks to contribute to the optimization of training programs, ultimately aiming to enhance clinical outcomes for patients with suspected ACS (Kok et al. 2016; Hart and Staveland 1988; Wood et al. 2013).

Training programs should address the cognitive challenges faced by novices, such as heightened mental workload and stress during ECG interpretation. Incorporating stress management techniques and simulations that gradually increase in complexity could help trainees adapt to the cognitive demands of real‐world scenarios in the ED. By integrating these elements, educators can accelerate the development of expert‐like performance in ECG interpretation, improving the diagnostic accuracy and efficiency of future cardiologists (Bond et al. 2014; Breen et al. 2014; Wood et al. 2013). Future intervention studies should aim to verify whether real‐time feedback can indeed shorten the learning curve by helping novices develop more targeted visual search strategies and manage cognitive load more effectively. Additionally, by simulating real‐world scenarios that gradually increase in complexity, educators can help trainees build resilience to cognitive load, preparing them for the high‐pressure demands of the ED and intensive care units.

### 4.5 Study Limitations

This pilot study has several limitations that should be considered when interpreting the results. First, the small sample size of eight participants, each representing a different level of expertise, constrains the generalizability of our findings. Larger cohorts are needed to verify whether these results extend to a broader population of cardiology practitioners. Additionally, the study was conducted in a controlled environment rather than a real‐world emergency department setting, which may limit the external validity of the results. In a clinical context, factors such as time pressure, patient interaction, and multitasking could influence interpretation strategies differently (Salerno et al. 2003; Fanaroff et al. 2018; Benjamin et al. 2019).

Another limitation is the reliance on eye‐tracking data as a proxy for cognitive load. While pupil dilation provides valuable insights into cognitive effort, it may also be influenced by other factors such as ambient lighting or emotional states, which were not controlled for in this study. Future studies could benefit from integrating other physiological measures, such as heart rate variability, to complement pupil dilation data and provide a more comprehensive understanding of cognitive load (Schläpfer and Wellens 2017; Wathen et al. 2019; Novotny et al. 2020). Lastly, the difficulty levels of the ECGs were determined based on expert consensus, which, while clinically relevant, may introduce subjectivity. Variability in difficulty perception among less experienced interpreters could influence their cognitive response to each ECG. Future research could explore more standardized methods for categorizing ECG difficulty and investigate how different case complexities influence learning and cognitive strategies over time (Thygesen et al. 2018; Zègre‐Hemsey et al. 2012).

Despite these limitations, the findings of this preliminary investigation offer meaningful insights into the cognitive and visual underpinnings of ECG interpretation expertise. These observations provide a foundation for refining targeted educational strategies within cardiology residency programs. Further research, carefully designed to overcome current methodological constraints, will be essential to determine how best to translate these observations into effective, evidence‐based training curricula, thereby facilitating the development of clinical proficiency among future cardiologists (Gegenfurtner et al. 2011; Sibbald et al. 2013; Waechter et al. 2019).

## 5 Conclusions

This preliminary investigation offers new insights into the cognitive foundations of visual expertise in ECG interpretation. Eye‐tracking analysis demonstrated that experienced cardiologists not only perform interpretations with superior speed and accuracy but also engage in distinct visual behaviors, characterized by fewer and longer fixations, reflecting enhanced cognitive efficiency and lower cognitive load as shown by reduced pupil dilation. Increased task complexity amplified these expertise‐related differences, disproportionately challenging less experienced interpreters. These findings highlight promising avenues for integrating eye‐tracking feedback and tailored cognitive strategies into cardiology residency training. Future research should evaluate whether these educational interventions can meaningfully accelerate skill acquisition and support trainees in achieving rapid diagnostic proficiency.

## Author Contributions

R.P., A.B., S.G. conceptualization, methodology, validation, and formal analysis were used to collect the data. A.B., C.C., F.C. validation, software, and data curation. A.B., F.R., M.P. writing, review, and editing. S.G., G.R. supervision and writing – review and editing. All authors contributed substantially to the original drafting and revisions. A.B. takes responsibility for the paper as a whole.

## Disclosure

The authors have nothing to report.

## Ethics Statement

IRB 254/2017.

## Conflicts of Interest

The authors declare no conflicts of interest.

## Supporting information


Tables

Items

## Data Availability

Data are available on request from the authors.
