# Supplementary material for: Tracing Visual Expertise in ECG Interpretation: An Eye‐Tracking Pilot Study
Source: Ann Noninvasive Electrocardiol. 2025 Apr 18;30(3):e70082. doi: 10.1111/anec.70082 (PMC12007014; doi:10.1111/anec.70082)
Supplement: Supplementary file 1 — Tables [file ANEC-30-e70082-s001.docx]

General information

| ID | Gender | Age Experience | |
| --- | --- | --- | --- |
| 2S | F | 27 | 2’ Year |
| 2Q | F | 52 | Expert |
| 1Q | M | 39 | Expert |
| 2T | F | 28 | 3’ Year |
| 1T | M | 32 | 3’ Year |
| 1S | M | 29 | 2’ Year |
| 1P | M | 28 | 1’ Year |
| 2P | F | 25 | 1’ Year |

Item list

| Item | Difficulties |
| --- | --- |
| ECG1 | Easy |
| ECG2 | Intermediate |
| ECG3 | Easy |
| ECG4 | Difficult |
| ECG5 | Easy |
| ECG6 | Difficult |
| ECG7 | Intermediate |
| ECG8 | Intermediate |
| ECG9 | Difficult |
