# Supplementary figures and images for: Tracing Visual Expertise in ECG Interpretation: An Eye‐Tracking Pilot Study
Source: Ann Noninvasive Electrocardiol. 2025 Apr 18;30(3):e70082. doi: 10.1111/anec.70082 (PMC12007014; doi:10.1111/anec.70082)

# ECG 1

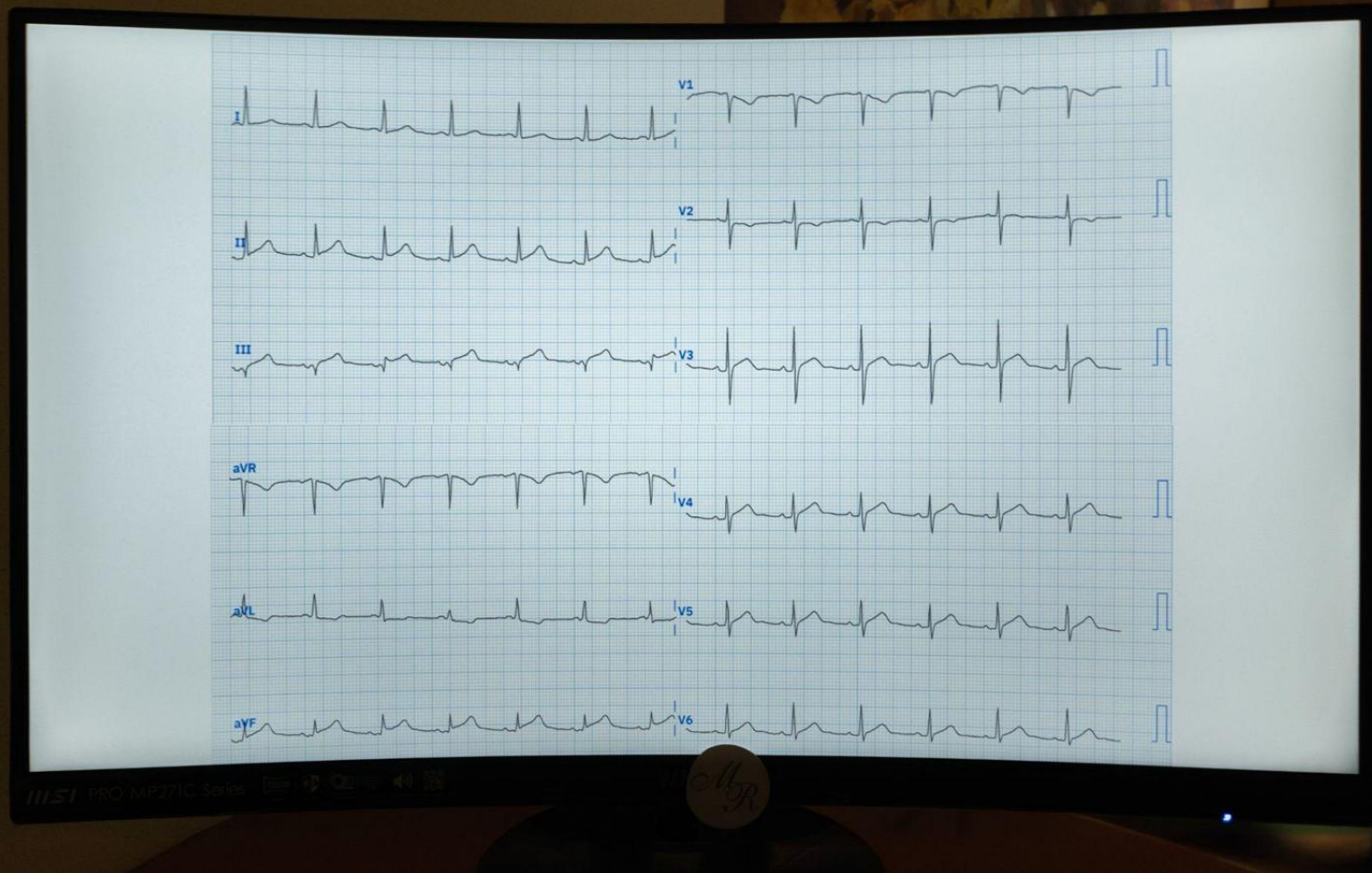

## ECG 2

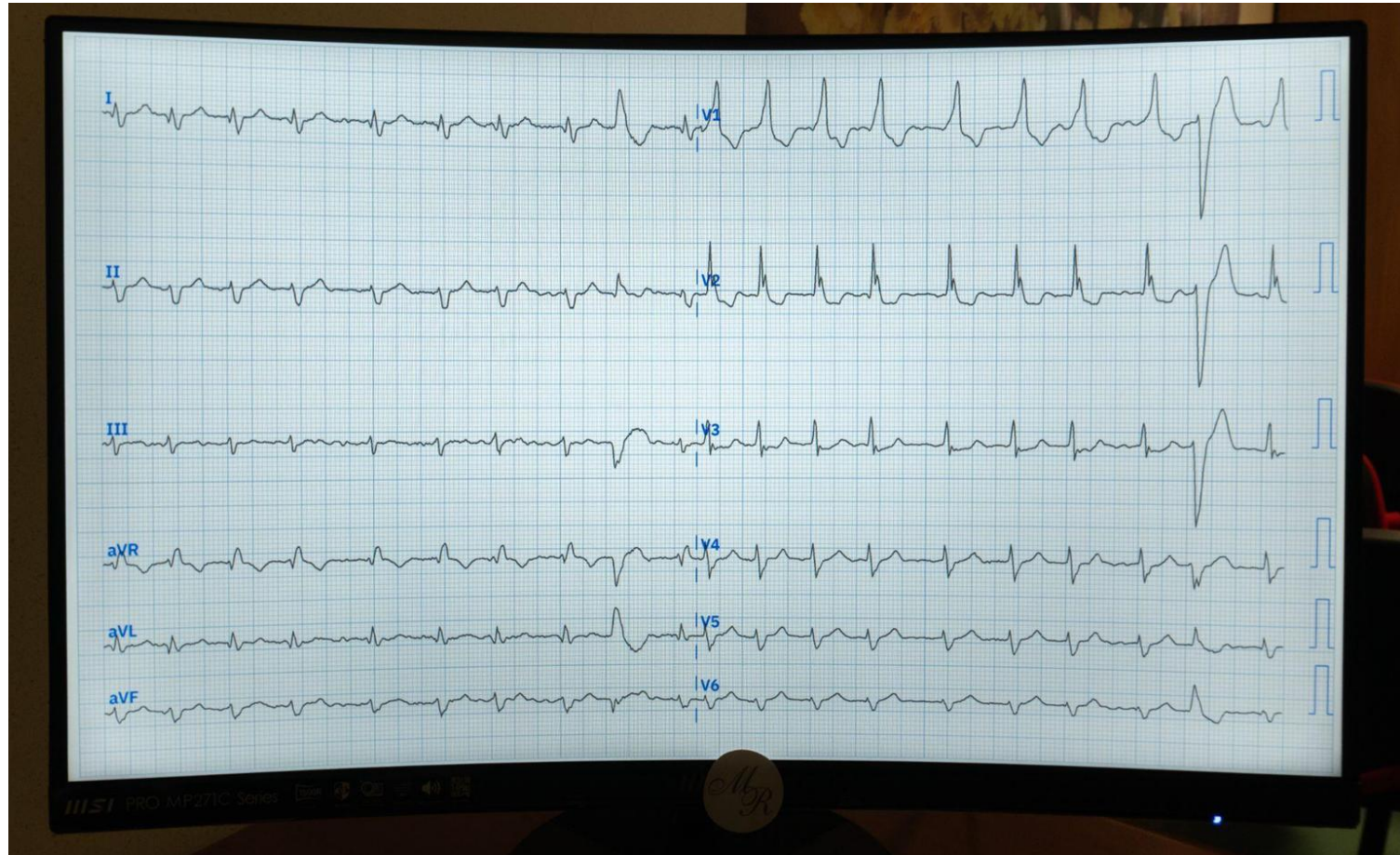

# ECG 3

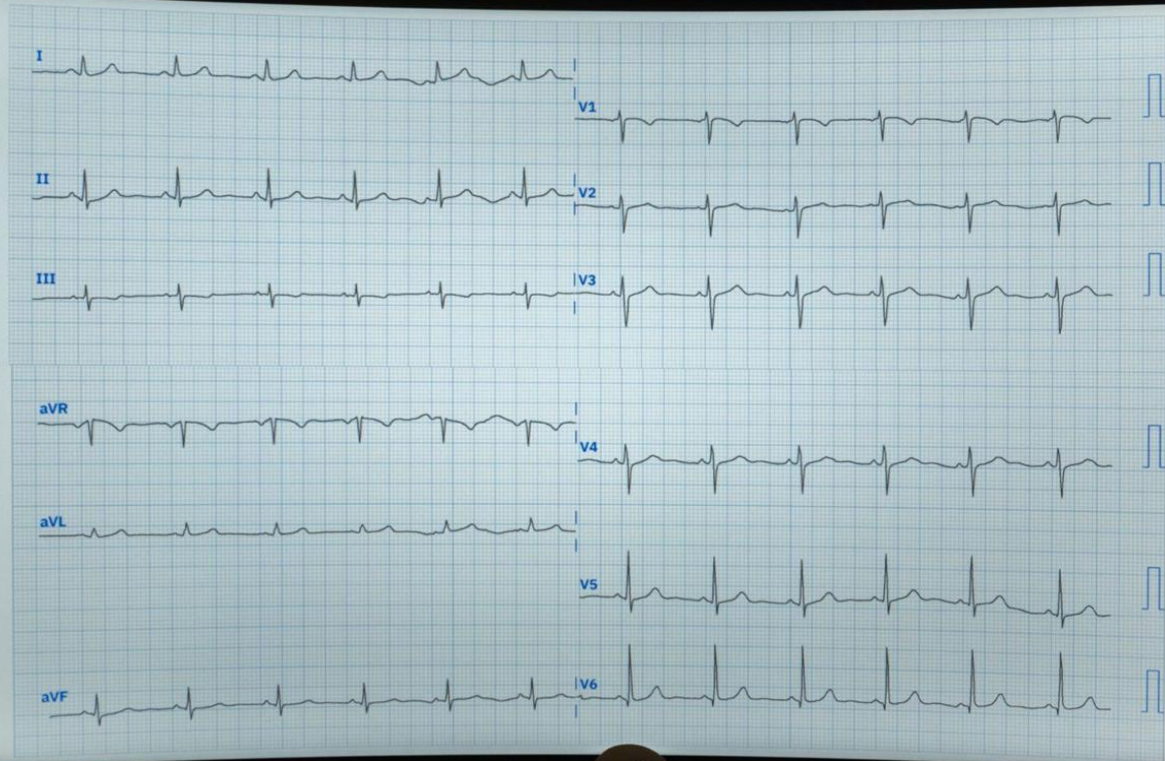

168

## ECG 4

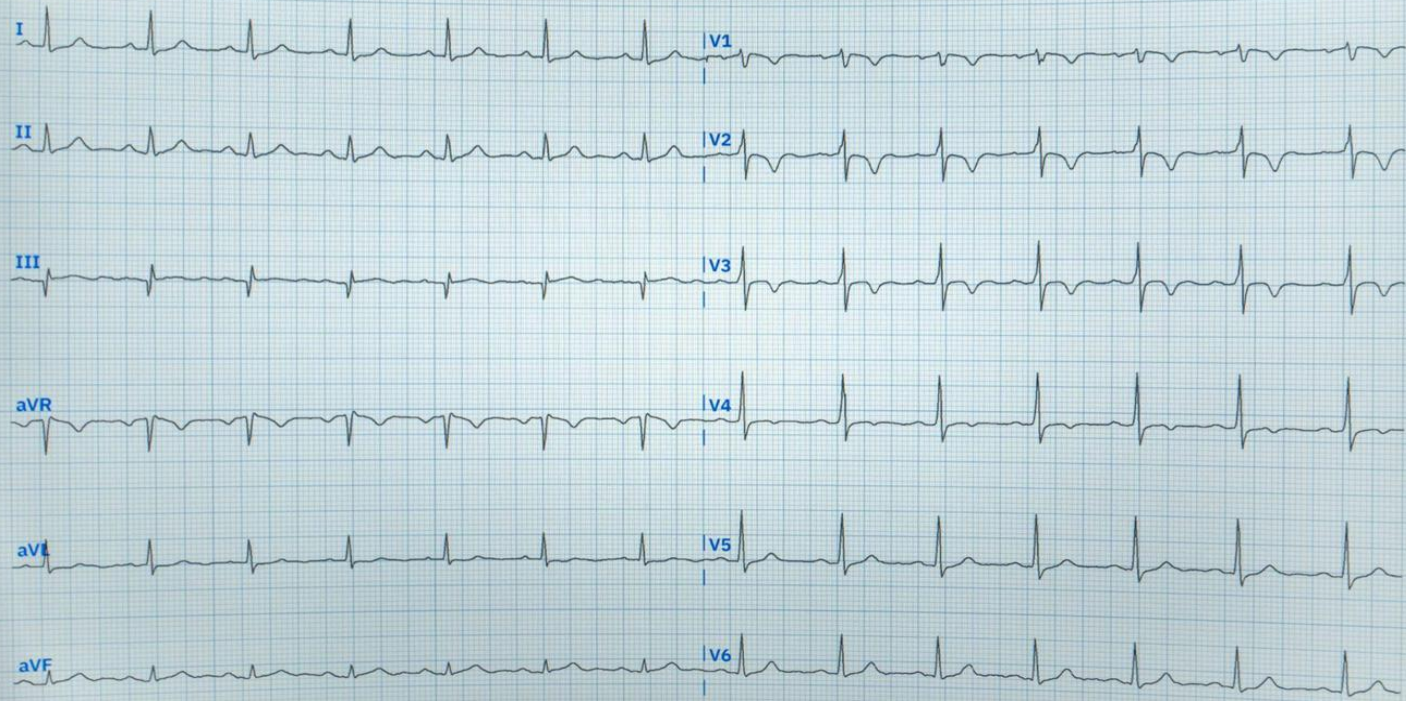

## ECG 5

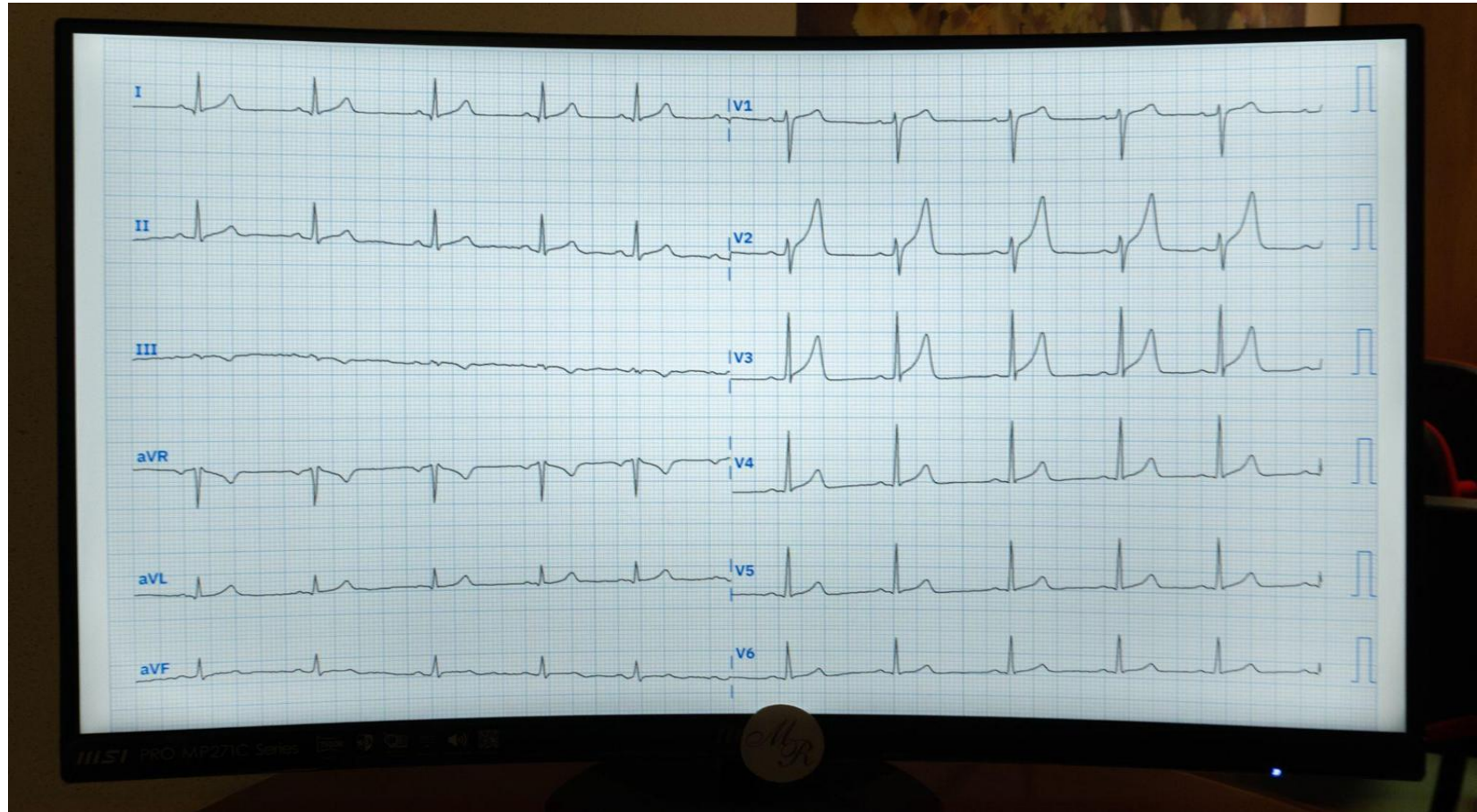

## ECG 6

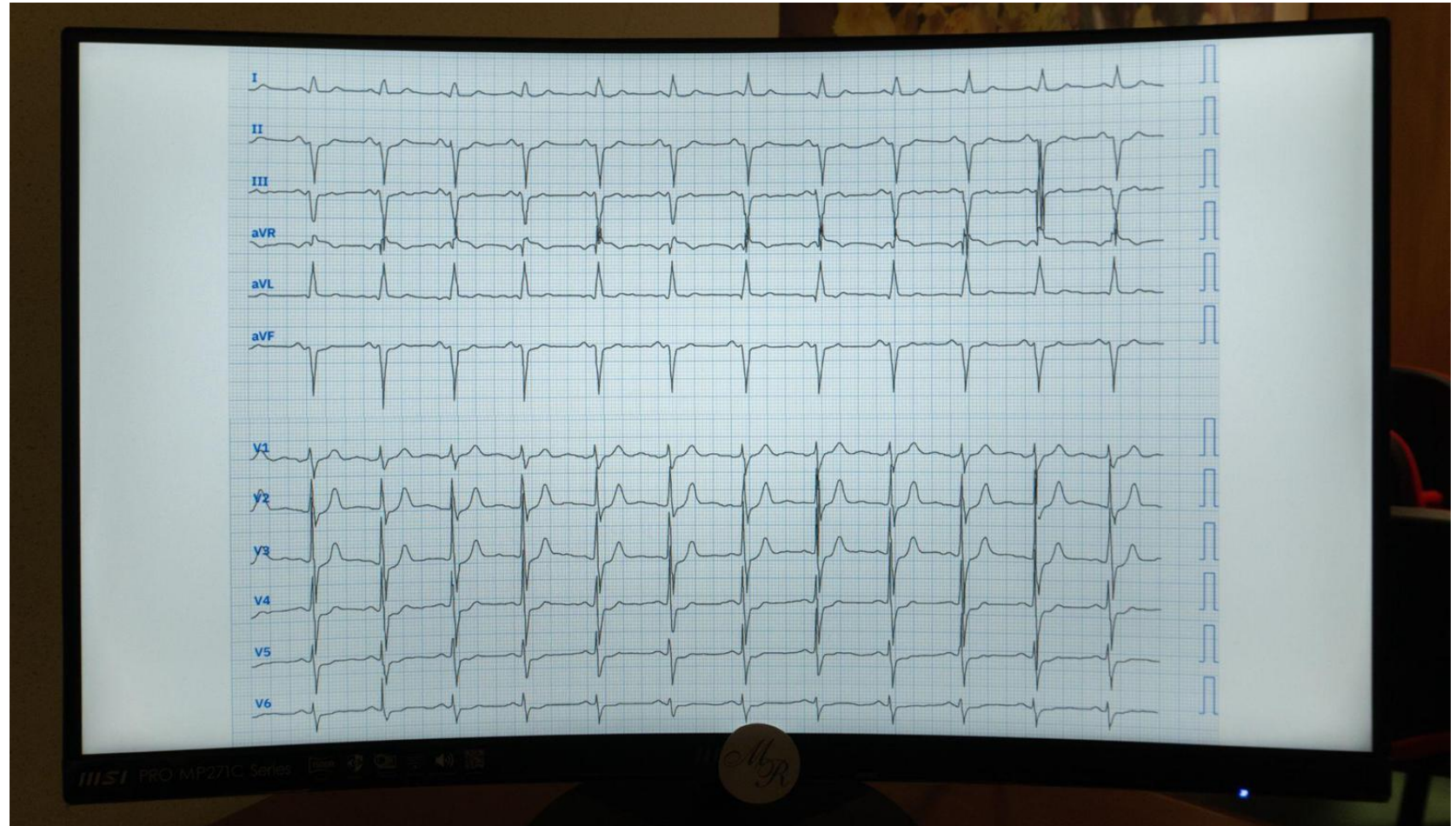

# ECG 7

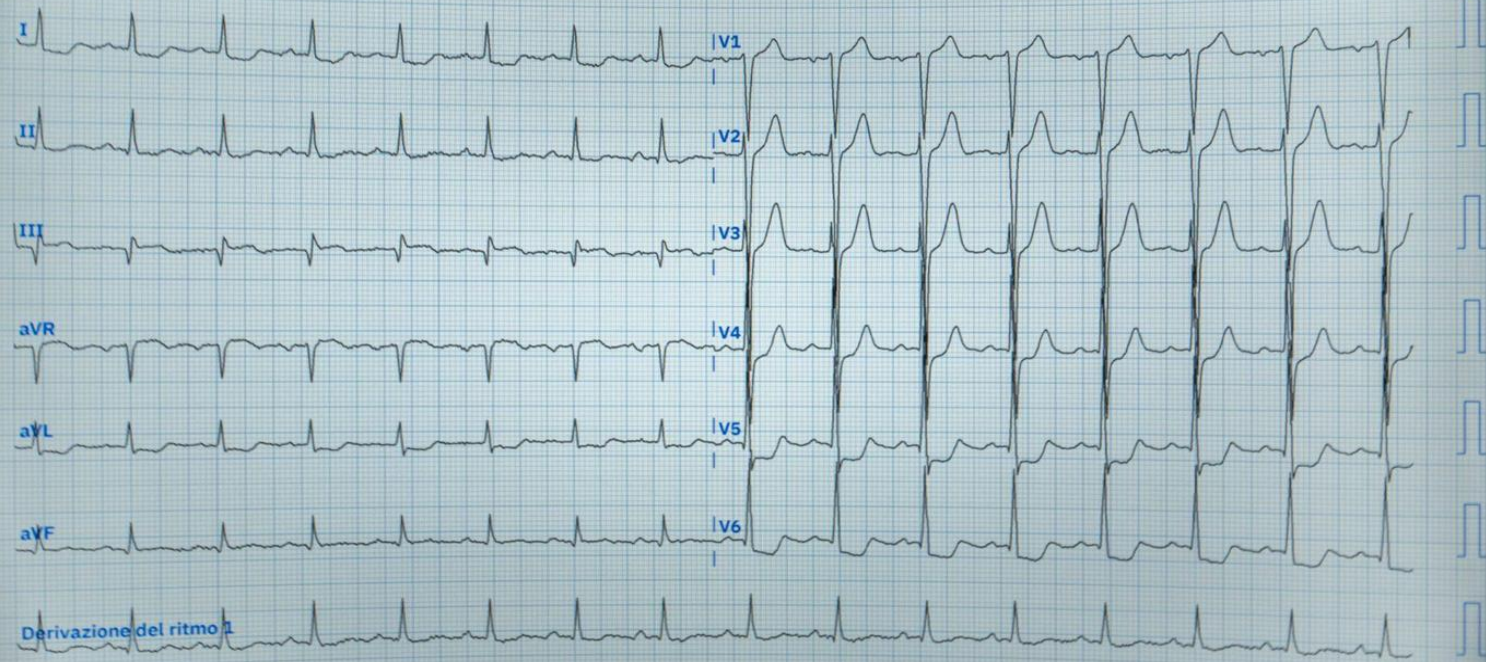

16R

## ECG 8

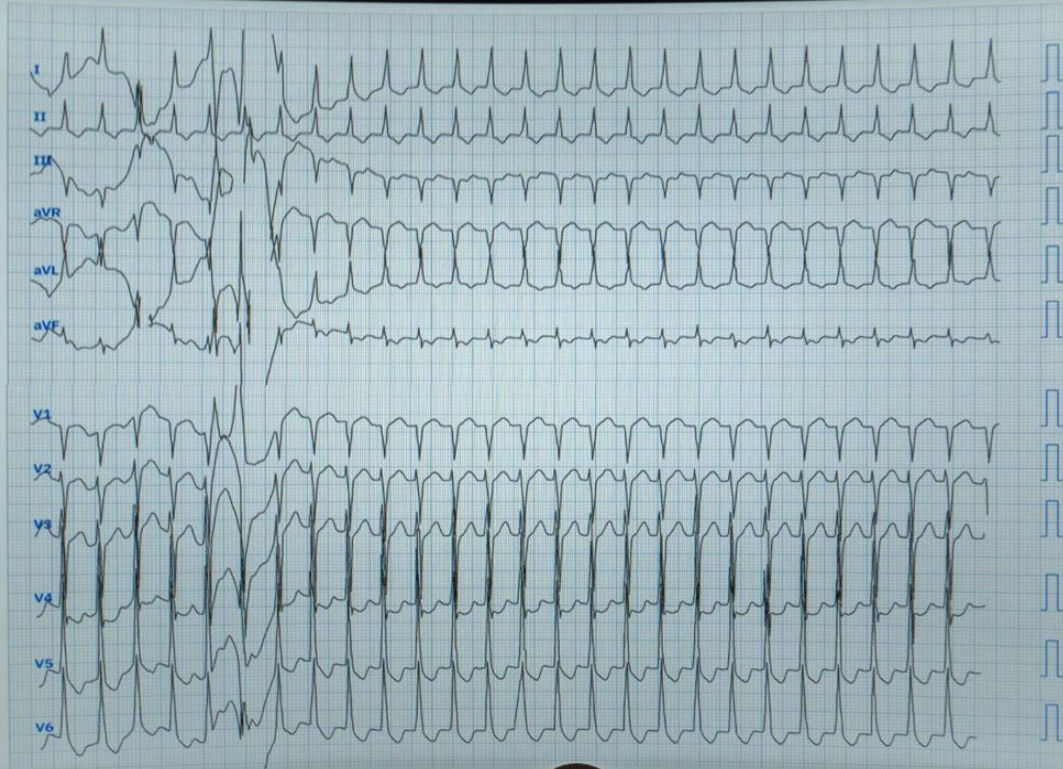

## ECG 9

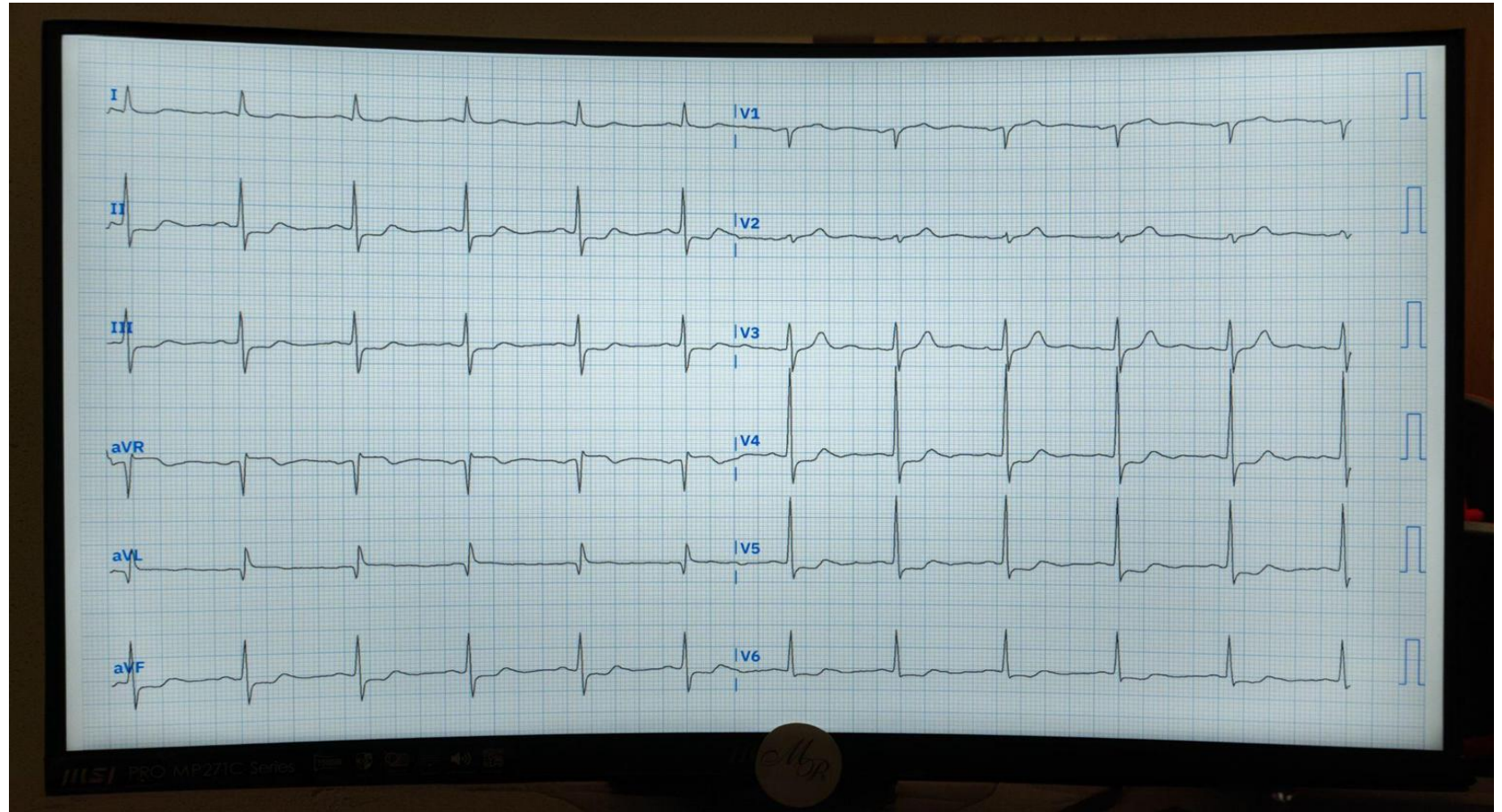

Supplement: Supplementary file 2 — Items [file ANEC-30-e70082-s002.pdf]
